# Primary Encapsulating Peritoneal Sclerosis: An Extremely Rare Cause of Small Bowel Obstruction

**DOI:** 10.7759/cureus.84065

**Published:** 2025-05-13

**Authors:** Faisal F Alnazawi, Aseel T Bnqadim, Sumayyah M Chapra

**Affiliations:** 1 General Surgery, International Medical Center, Jeddah, SAU; 2 Surgery, King Abdulaziz University Hospital, Jeddah, SAU; 3 General Surgery, King Fahad General Hospital, Jeddah, SAU

**Keywords:** abdominal cocoon syndrome, diagnosis, encapsulating peritoneal sclerosis, small bowel obstruction, surgical management

## Abstract

Encapsulating peritoneal sclerosis (EPS) is a rare condition characterized by a fibrocollagenous membrane encasing the small intestine, leading to bowel obstruction. We report a case of a 31-year-old healthy male presenting with chronic abdominal pain, distention, weight loss, and jejuno-ileal intussusception managed conservatively at another facility. Despite hydration and bowel rest, symptoms persisted, prompting surgical intervention. A thick fibrous capsule encased the small bowel intraoperatively, necessitating adhesiolysis and capsule excision. The appendix was also removed prophylactically. Postoperatively, the patient experienced prolonged paralytic ileus, managed successfully with total parenteral nutrition and supportive measures. Histopathology confirmed collagenous peritoneal thickening with inflammatory infiltrates. The patient recovered well and is regaining bowel function; follow-ups showed no recurrence. This case highlights the diagnostic challenges of EPS, emphasizing the role of computed tomography and the need for surgical intervention in advanced cases to prevent complications. Early recognition and management are crucial for favorable outcomes.

## Introduction

Encapsulating peritoneal sclerosis (EPS) is an uncommon medical condition marked by the development of an inflammatory fibrocollagenous membrane around the small intestine. This results in symptoms of bowel obstruction [1]. The International Society for Peritoneal Dialysis defines EPS as a syndrome characterized by the recurrent or continuous manifestation of intestinal obstruction symptoms due to adhesions formed by a thickened peritoneum [2]. This condition predominantly affects patients with chronic peritoneal irritation and inflammation, notably those undergoing long-term peritoneal dialysis for end-stage renal disease; however, it can also arise secondary to intra-abdominal infections (e.g., tuberculosis), previous abdominal surgeries, peritoneal carcinomatosis, and certain medications (e.g., beta-blockers) [3]. Patients typically exhibit abdominal symptoms accompanied by inflammatory indicators, such as fever, elevated levels of C-reactive protein, and blood-stained ascites in the early phases. Later in the advanced stages of the disease, more pronounced clinical features emerge, primarily due to ileus and/or the formation of peritoneal adhesions [4]. Currently, no laboratory tests are available for EPS, and radiographic imaging is the recommended approach to confirm the diagnosis, with CT being the definitive imaging method for EPS [5]. In equivocal cases or to obtain histologic confirmation, diagnostic laparoscopy, performed cautiously due to adhesion risks, can visualize the fibrocollagenous membrane. However, a definitive diagnosis requires biopsy and histopathological examination, demonstrating fibrocollagenous thickening and inflammatory infiltrate [6]. Management is tailored to disease severity. In early or mild-to-moderate EPS, medical interventions aim to attenuate inflammation and fibrosis: corticosteroids and tamoxifen have shown efficacy in arresting disease progression, often in combination with nutritional support. If medical intervention fails, surgery may be necessary [7]. Due to the rarity of the condition and its associated morbidity, we present a case of EPS in a 31-year-old medically and surgically free patient. We operated on the patient and obtained a satisfactory result.

## Case presentation

**Figure 1:**
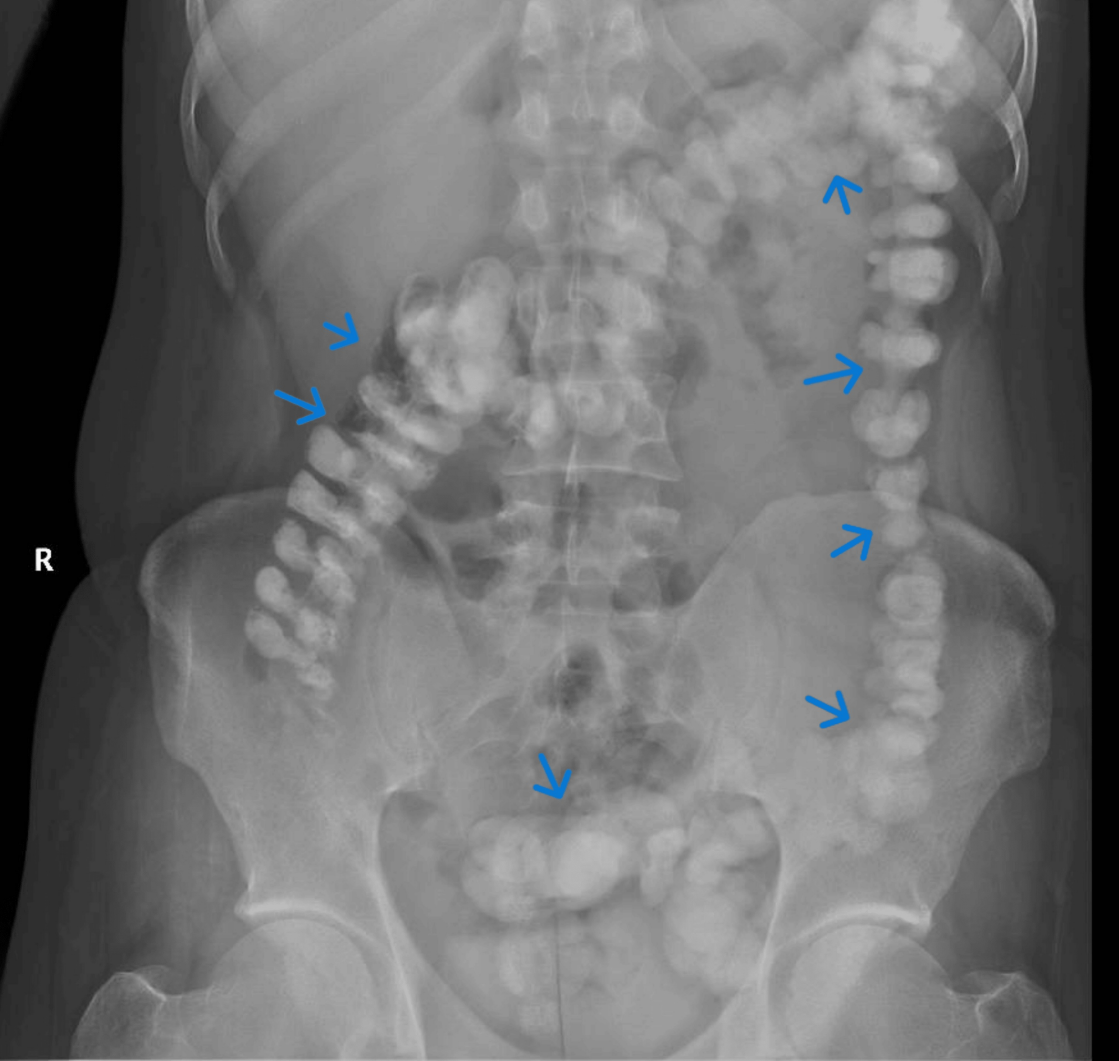
Erect abdominal X-ray done upon admission showing colon filled with stool and stool impaction in the sigmoid (blue arrows indicate stool impaction)

A 31-year-old male with no known medical or surgical history presented with recurrent abdominal pain of prolonged duration, associated with persistent abdominal distention for the past five days. He reported an unintentional weight loss of 9 kg over the preceding six months. He denied any history of fever, night sweats, lymphadenopathy, hematemesis, melena, or skin discoloration. The patient also denied any previous similar episodes, personal or family history of tuberculosis, or known contact with individuals diagnosed with tuberculosis. A CT scan at another facility revealed a jejuno-ileal intussusception, which was managed conservatively. On examination, the patient appeared well, but he does have abdominal distention and tenderness in the epigastric area. His blood tests were within the normal ranges (Table 1). An erect abdominal X-ray on admission showed the colon filled with stool, with impaction in the sigmoid (Figure 1). Consequently, he was admitted for repeat CT imaging and IV hydration.

**Table 1:** Laboratory blood tests on admission

Test	Result
White blood cell count	8.81 × 10^9^/L
Red blood cell count	5.67 × 10^9^/L
Hemoglobin	16.8 G/dl
Platelets	258 × 10^9^/L
Chloride	101 Meq/L
Sodium	141 Meq/L
Blood urea nitrogen	9.6 Mg/dl
Potassium	4.56 Meq/L
Calcium	9.7 Mg/dl
Creatinine	0.69 Mg/dl
Aspartate aminotransferase	40.7 U/L
Alanine aminotransferase	35.8 U/L
Gamma-glutamyl transferase	52 U/L
Total bilirubin	0.17 Mg/dl
Albumin	3.8 G/dl
Phosphorous	4.05 Mg/dl

**Figure 2:**
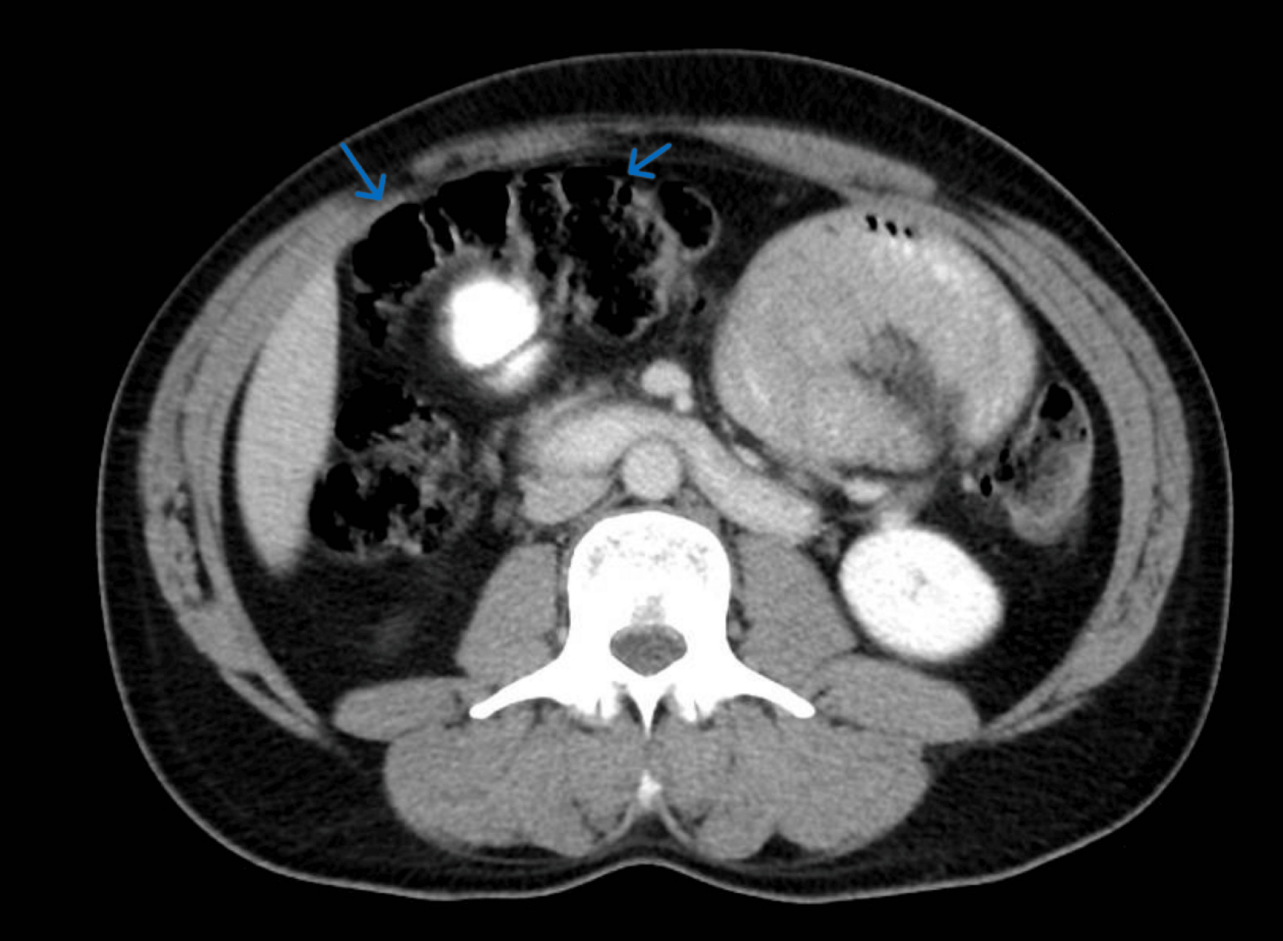
CT of the abdomen and pelvis with oral and IV contrast showing a clustering of small bowel loops likely encapsulated within a sac-like structure (blue arrows indicate small bowel loops) CT: computed tomography, IV: intravenous

Despite being kept NPO (none per oral) and receiving IV fluids for several days, symptoms persisted. On day 3 of admission, a contrast CT scan revealed encapsulated small bowel loops (Figure 2).

**Figure 3:**
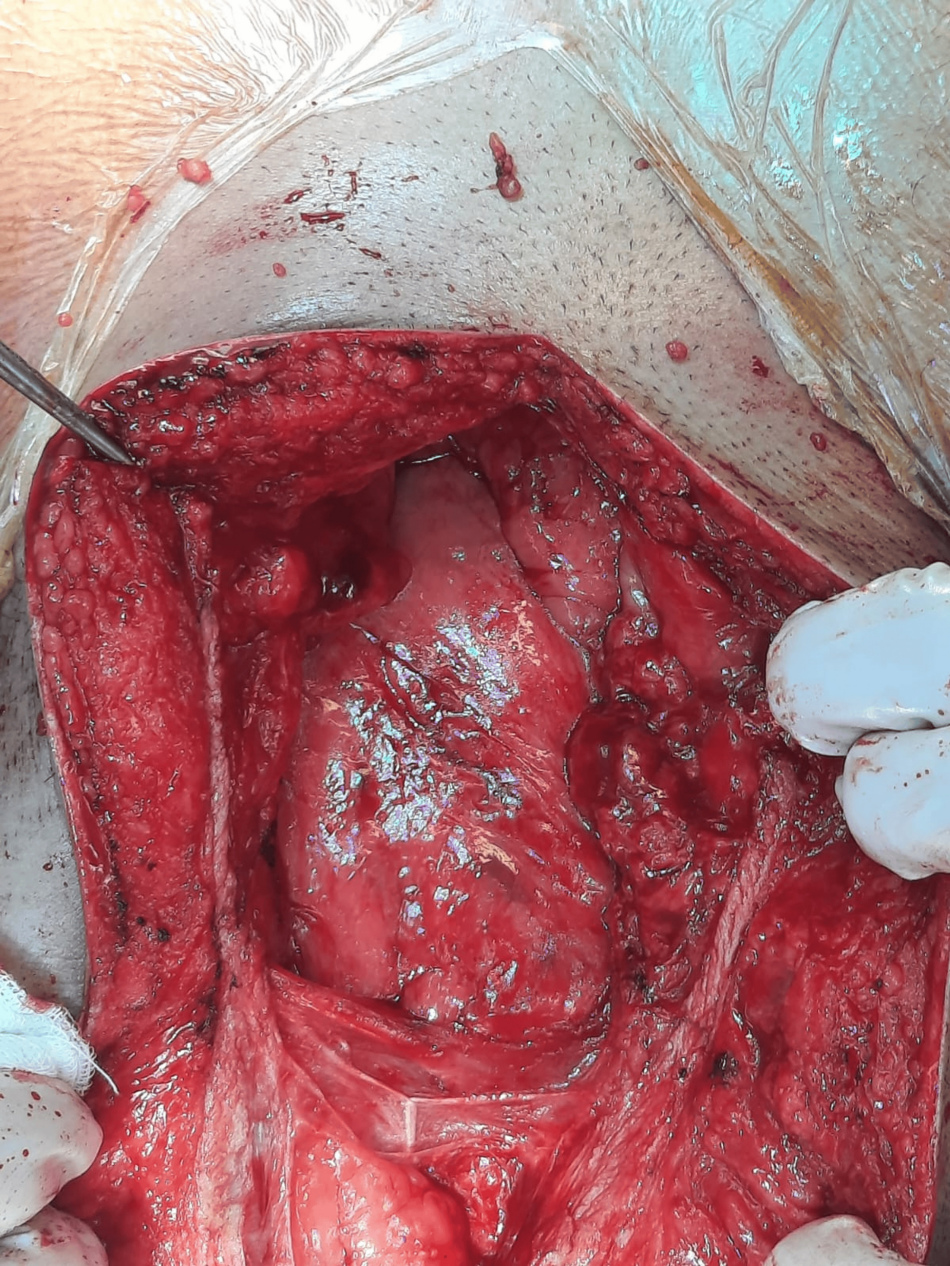
Thick membrane covering the small intestine is identified by entering the abdominal cavity

**Figure 4:**
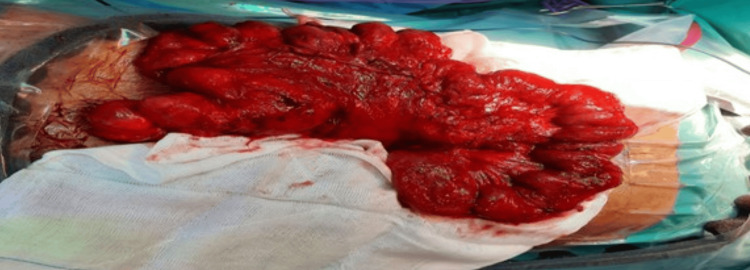
Surface of the small intestine is covered with a layer of fibrous membrane structure

**Figure 5:**
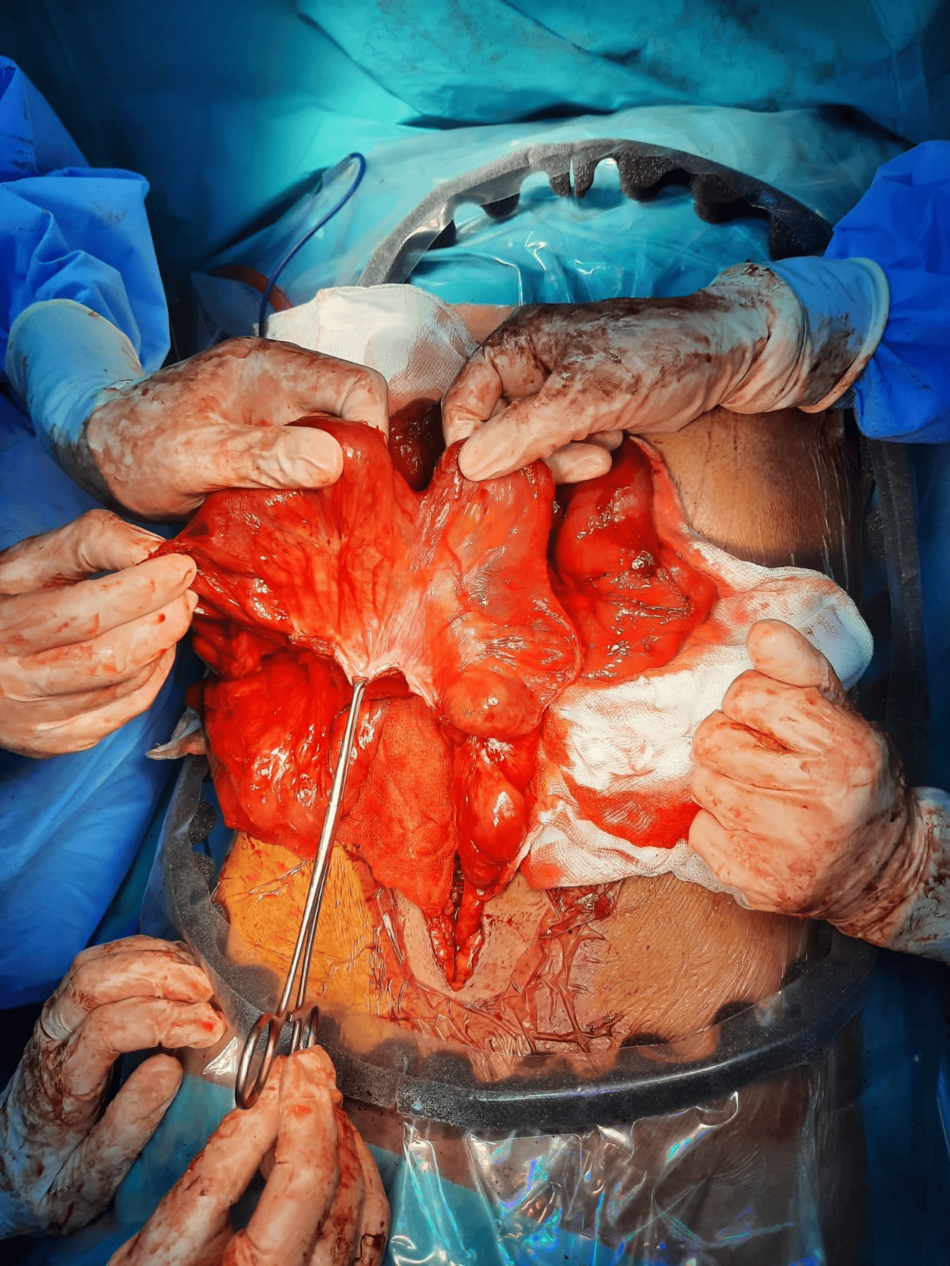
Membrane is being removed

After the failure of conservative management, the decision has been made to release the sac-like structure and examine the abdomen for abnormalities. The patient was prepared for the operation, and consent was obtained. Additionally, two units of packed red blood cells have been prepared in case of an emergency situation. Intraoperatively, the small bowel was found to be encapsulated with a thick fibrous capsule on the right and left sides of the abdomen, with adhesions within the capsule and no evidence of infectious or peritoneal fluid (Figures 3-4). We started laterally and opened the capsule near the abdominal wall, followed by adhesiolysis with the aid of diathermy until the small bowel was completely released (Figure 5). After running the small bowel from the distal jejunum to the ileocecal junction and confirming no remaining adhesions or encapsulation and no bowel injury, histopathology samples were obtained, and a prophylactic appendectomy was completed. His postoperative course was complicated by a prolonged paralytic ileus and treated with total parenteral nutrition, glycerin suppositories, and early mobilization. Repeat abdominal CT did not show any evidence of bowel injury or mechanical obstruction. By postoperative day 7, he passed stool, and bowel function normalized. Tumor markers (AFP, CEA, and CA 19-9) were all within normal ranges. The patient was seen in the outpatient clinic one week and one month after discharge. He was doing well with a healed wound and improvement in symptoms. He was informed that he may develop adhesive intestinal obstruction in the future and will need to seek medical attention. He was advised to avoid lifting heavy objects for three months.

Final histopathology demonstrated reactive lymphoid hyperplasia of the appendix and a peritoneal membrane with a collagenous wall and focal mixed inflammatory infiltrate.

## Discussion

EPS was described in 1907 as “peritonitis chronica fibrosa incapsulata” [8]. Though the exact pathophysiology of EPS is unknown, it is thought to result from activation of fibroblasts and release of cytokines during peritoneal inflammation [9]. In a study by Akbulut, 193 documented cases were analyzed, with findings revealing that EPS occurs twice as frequently in men compared to women, which contradicts theories suggesting retrograde menstruation as a potential etiology [10]. The exact etiology of EPS remains unknown. The preoperative diagnosis continues to pose a significant challenge owing to the manifestation of nonspecific ileus-like symptoms [4]. Hence, preoperative diagnostic strategies necessitate a heightened level of clinical suspicion, particularly discernible from recurrent non-strangulating bowel obstruction episodes, distinctive radiological observations, and the absence of other identifiable causes [4,6]. The case discussed in this study illustrates the acute onset of EPS. While patient history, physical examination, lab tests, and X-ray were all nonspecific, the abdominal CT scan was the only reliable modality for an accurate diagnosis. The management of EPS is debatable, with many clinicians opting for surgical intervention. However, surgery should be avoided in mild and asymptomatic cases to avoid postoperative iatrogenic complications. In instances where diagnosis occurs late in the disease progression and there is partial or complete small bowel obstruction, as in our case, the authors conclude that surgical management is warranted. The surgical approach to treating EPS follows principles that involve an exploratory laparotomy aimed at completely excising the peritoneal capsule and adhesiolysis. Prophylactic appendectomy, as performed in our case, has been suggested by some authors to avoid complicated appendectomy in the future [10,11].

## Conclusions

EPS remains a rare but potentially serious condition with significant diagnostic and therapeutic challenges. This case highlights the importance of considering EPS in patients presenting with recurrent or unexplained bowel obstruction, even in the absence of typical risk factors such as peritoneal dialysis. While imaging, particularly CT, is crucial in raising suspicion, definitive diagnosis and management often rely on surgical exploration. Timely intervention, including adhesiolysis and excision of the fibrous capsule, can result in favorable outcomes. Increased clinical awareness and documentation of such atypical presentations are essential to enhance early recognition and guide optimal management strategies.
